# Supplementary material for: The minimal intrinsic stochasticity of constitutively expressed eukaryotic genes is sub-Poissonian
Source: bioRxiv. 2023 Mar 8:2023.03.06.531283. Preprint. [Version 2] doi: 10.1101/2023.03.06.531283 (PMC10028819; doi:10.1101/2023.03.06.531283)
Supplement: Supplement 1 [file media-1.pdf]

Supplementary Information

Table of Contents

Section A: Statistical tests of size-corrected Fano factors 2

A.1 Statistical methodology 2

A.2 Comparison of hierarchical bootstrapping with bootstrapping from pooled data 2

A.3 Comparison of Fano factors in cellular compartments 4

A.4 Comparison of Fano factors between mononucleated and binucleated cells 6

A.5 Comparison of Fano factors between endogenous and exogenous locus and with different coding sequences 7

Section B: Modeling and Inference 8

B.1 Model specification 8

B.2 General properties of the model 9

B.3 Bayesian model selection and parameter inference 11

B.4 Testing the ansatz of Poisson fluctuations 21

Tables S1 – S4 23

Section A: Statistical tests of size-corrected Fano factors

A.1 Statistical methodology

For statistical conclusions on the size-corrected Fano factors, we used a hierarchical bootstrapping approach. Experimental replicates (shown separately in the main figures) and cells within replicates were resampled with replacement. From this, the statistic of interest (average Fano factor across experimental replicates, difference between genotypes, average difference in Fano factor between mono- and binucleated cells, or average difference in Fano factor between cellular compartments) was calculated. To estimate the statistic's distribution, 5,000 bootstrap replicates were performed, and the mean and 95 % confidence interval (2.5th to 97.5th percentile) were calculated from the bootstrap results. Up to 0.5 % of bootstrap replicates were allowed to fail to calculate (i.e., returned NaN), and these were discarded before calculating the mean and confidence interval. If > 0.5 % of bootstrap replicates failed, no statistical results were reported for that analysis. The latter was only the case for mature nuclear mRNA from *mad1* in binucleated cells (TS-labelled data), due to a low cell number in one replicate. A genotype was considered to have a size-corrected RNA distribution significantly different from Poisson if the bootstrap 95 % confidence interval excluded 1, and differences were considered significant if the confidence interval excluded 0.

A.2 Comparison of hierarchical bootstrapping with bootstrapping from pooled data

As a comparison to hierarchical bootstrapping, cells from replicate experiments were combined and the cell-size corrected Fano factor calculated from the pooled set of cells. This yielded very similar results to the hierarchical bootstrapping results (Fig. A-I, A-III), except for some genes in published data (*1*, *2*) with a larger variation between experimental replicates (Fig. A-II).

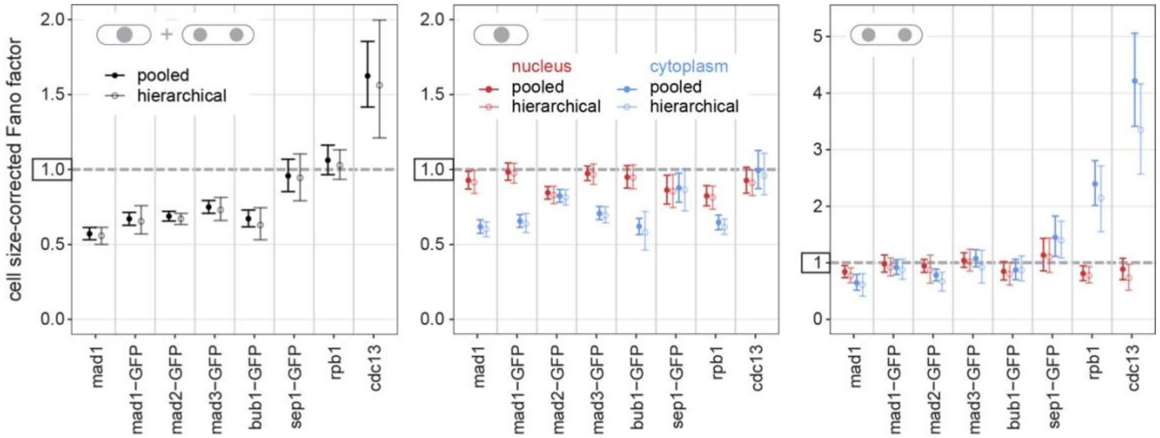

Figure A-I: Fano factors from pooled and hierarchical analysis (non-TS labelled)

Related to Fig. 2 and Fig. 6F and G.

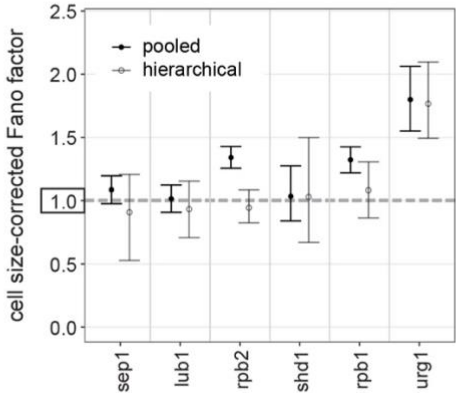

Figure A-II: Fano factors from pooled and hierarchical analysis of Marguerat group data

Related to Fig. 2D. Only genes with more than one experimental replicate are shown.

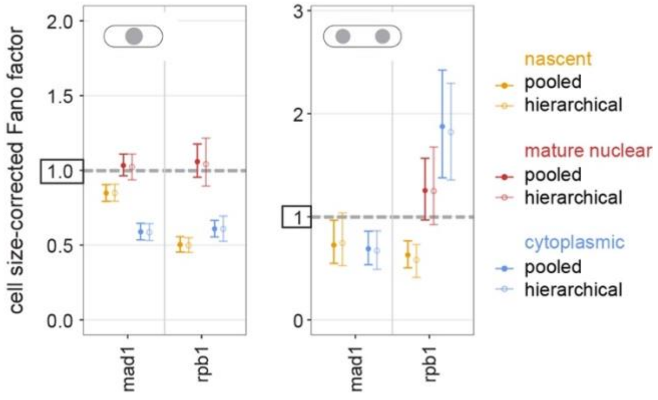

Figure A-III: Fano factors from pooled and hierarchical analysis (TS-labelled)

Related to Fig. 7E.

A.3 Comparison of Fano factors in cellular compartments

We tested if size-corrected Fano factors differ between cellular compartments using hierarchical bootstrapping. Mean and 95 % confidence intervals from bootstrapping the mean difference between compartments are provided. Confidence intervals that exclude 0 are interpreted as significantly different Fano factors between compartments.

Table A-I: Comparison between nuclear and cytoplasmic Fano factors (non-TS labelled)

Comparing nuclear and cytoplasmic Fano factors in experiments without transcription-site labelling (see Fig. 6 and A-I). A positive mean Fano factor difference indicates a larger Fano factor in the cytoplasm than the nucleus, while a negative mean Fano factor difference indicates a smaller Fano factor in the cytoplasm than the nucleus.

Gene	Nuclei Count	Mean Fano Factor Difference	Lower 95 % Confidence Interval	Upper 95 % Confidence Interval	Effect
mad1	1	-0.31	-0.39	-0.23	smaller
mad1	2	-0.16	-0.38	0.06	same
mad1	all	-0.29	-0.37	-0.20	smaller
mad1-GFP	1	-0.34	-0.42	-0.25	smaller
mad1-GFP	2	-0.04	-0.25	0.18	same
mad1-GFP	all	-0.29	-0.37	-0.20	smaller
mad2-GFP	1	-0.02	-0.09	0.07	same
mad2-GFP	2	-0.20	-0.52	0.05	same
mad2-GFP	all	-0.04	-0.10	0.03	same
mad3-GFP	1	-0.27	-0.37	-0.18	smaller
mad3-GFP	2	-0.11	-0.53	0.26	same
mad3-GFP	all	-0.22	-0.33	-0.12	smaller
bub1-GFP	1	-0.37	-0.52	-0.20	smaller
bub1-GFP	2	0.07	-0.25	0.42	same
bub1-GFP	all	-0.26	-0.38	-0.13	smaller
sep1-GFP	1	0.01	-0.13	0.15	same
sep1-GFP	2	0.29	-0.11	0.69	same
sep1-GFP	all	0.06	-0.11	0.22	same
rpb1	1	-0.20	-0.29	-0.10	smaller
rpb1	2	1.37	0.72	1.99	larger
rpb1	all	0.18	0.05	0.30	larger
cdc13	1	0.05	-0.10	0.22	same
cdc13	2	2.62	1.90	3.37	larger
cdc13	all	0.66	0.30	1.09	larger
SPAC2H10.01-GFP	1	1.88	0.79	3.05	larger
SPAC2H10.01-GFP	2	1.31	0.61	1.95	larger
SPAC2H10.01-GFP	all	1.97	0.91	3.05	larger
SPAC27D7.09c-GFP	1	6.84	4.82	9.03	larger
SPAC27D7.09c-GFP	2	3.19	-1.93	7.89	same
SPAC27D7.09c-GFP	all	6.63	4.74	8.67	larger

Table A-II: Comparison between nuclear and cytoplasmic Fano factors at the exogenous wis1 locus

Comparing nuclear and cytoplasmic Fano factors at the exogenous wis1 locus (see Fig. 5E). A positive mean Fano factor difference indicates a larger Fano factor in the cytoplasm than the nucleus, while a negative mean Fano factor difference indicates a smaller Fano factor in the cytoplasm than the nucleus. Change observed in mononucleated cells (as opposed to mononucleated and binucleated cells pooled = all) is indicated in the second effect column, without listing the mean Fano factor difference and confidence intervals.

Gene	Mean Fano Factor Difference	Lower 95 % Confidence Interval	Upper 95 % Confidence Interval	Effect all	Effect mono-nucl.
mad2 endog	-0.04	-0.11	0.03	same	same
mad3 endog	-0.23	-0.33	-0.12	smaller	smaller
rad21 endog	6.74	5.82	7.71	larger	larger
mad2 exog	0.02	-0.12	0.16	same	same
Pmad2-rad21 exog	0.24	0.02	0.46	larger	same
Pmad2-nmt1 exog	0.10	-0.06	0.26	same	same
mad3 exog	0.03	-0.12	0.17	same	same
Pmad3-rad21 exog	0.50	0.27	0.73	larger	same
Pmad3-nmt1 exog	0.26	0.03	0.49	larger	same

Table A-III: Comparison of Fano factors in different compartments (TS-labelled)

Comparing nascent, mature nuclear, and cytoplasmic Fano factors in experiments with transcription-site labelling (see Fig. 7 and A-III). A positive mean Fano factor difference indicates a larger Fano factor in compartment 2 than in compartment 1, while a negative mean Fano factor difference indicates a smaller Fano factor in compartment 2 than in compartment 1.

Gene	Nuclei Count	Compartment 1	Compartment 2	Mean Fano Factor Difference	Lower 95 % Confidence Interval	Upper 95 % Confidence Interval	Effect
mad1	1	Nascent	Mature Nuclear	0.17	0.07	0.28	larger
mad1	1	Nascent	Cytoplasm	-0.26	-0.34	-0.18	smaller
mad1	1	Mature Nuclear	Cytoplasm	-0.44	-0.54	-0.33	smaller
mad1	2	Nascent	Mature Nuclear	N/A	N/A	N/A	N/A
mad1	2	Nascent	Cytoplasm	-0.08	-0.45	0.23	same
mad1	2	Mature Nuclear	Cytoplasm	N/A	N/A	N/A	N/A
mad1	all	Nascent	Mature Nuclear	0.20	0.07	0.31	larger
mad1	all	Nascent	Cytoplasm	-0.23	-0.32	-0.15	smaller
mad1	all	Mature Nuclear	Cytoplasm	-0.42	-0.52	-0.32	smaller
rpb1	1	Nascent	Mature Nuclear	0.54	0.40	0.71	larger
rpb1	1	Nascent	Cytoplasm	0.11	0.02	0.21	larger
rpb1	1	Mature Nuclear	Cytoplasm	-0.43	-0.57	-0.31	smaller
rpb1	2	Nascent	Mature Nuclear	0.67	0.29	1.21	larger
rpb1	2	Nascent	Cytoplasm	1.23	0.75	1.72	larger
rpb1	2	Mature Nuclear	Cytoplasm	0.57	-0.14	1.19	same
rpb1	all	Nascent	Mature Nuclear	0.53	0.42	0.65	larger
rpb1	all	Nascent	Cytoplasm	0.48	0.28	0.70	larger
rpb1	all	Mature Nuclear	Cytoplasm	-0.06	-0.28	0.17	same

A.4 Comparison of Fano factors between mononucleated and binucleated cells

We tested if size-corrected Fano factors differ between mono- and binucleated cells using hierarchical bootstrapping. Mean and 95 % confidence intervals from bootstrapping the mean difference are provided. Confidence intervals that exclude 0 are interpreted as a significant difference in Fano factors.

Table A-IV: Comparison of Fano factors between binucleated and mononucleated cells (non-TS labelled)

Comparing Fano factors from mono- and binucleated cells in experiments without transcription-site labelling (see Fig. 6 and A-I). A positive mean Fano factor difference indicates a larger Fano factor in binucleated than mononucleated cells, while a negative mean Fano factor difference indicates a smaller Fano factor in binucleated than mononucleated cells.

Gene	Compartment	Mean Fano Factor Difference	Lower 95 % Confidence Interval	Upper 95 % Confidence Interval	Effect
mad1	Nucleus	-0.14	-0.27	-0.02	smaller
mad1	Cytoplasm	0.01	-0.21	0.22	same
mad1-GFP	Nucleus	-0.06	-0.22	0.12	same
mad1-GFP	Cytoplasm	0.24	0.08	0.41	larger
mad2-GFP	Nucleus	0.05	-0.22	0.33	same
mad2-GFP	Cytoplasm	-0.14	-0.34	0.04	same
mad3-GFP	Nucleus	0.06	-0.13	0.31	same
mad3-GFP	Cytoplasm	0.22	-0.04	0.52	same
bub1-GFP	Nucleus	-0.14	-0.37	0.09	same
bub1-GFP	Cytoplasm	0.29	0.06	0.57	larger
sep1-GFP	Nucleus	0.25	-0.06	0.61	same
sep1-GFP	Cytoplasm	0.53	0.21	0.88	larger
rpb1	Nucleus	-0.04	-0.20	0.15	same
rpb1	Cytoplasm	1.53	0.91	2.11	larger
cdc13	Nucleus	-0.18	-0.41	0.08	same
cdc13	Cytoplasm	2.38	1.61	3.19	larger
SPAC2H10.01-GFP	Nucleus	-0.93	-1.80	-0.13	smaller
SPAC2H10.01-GFP	Cytoplasm	-1.47	-2.57	-0.46	smaller
SPAC27D7.09c-GFP	Nucleus	3.87	1.08	6.16	larger
SPAC27D7.09c-GFP	Cytoplasm	0.19	-5.79	5.38	same

Table A-V: Comparison of Fano factors between binucleated and mononucleated cells (TS-labelled)

Comparing Fano factors from mono- and binucleated cells in experiments with transcription-site labelling (see Fig. 7 and A-III). A positive mean Fano factor difference indicates a larger Fano factor in binucleated than mononucleated cells.

**Gene**	**Compartment**	**Mean Fano Factor Difference**	**Lower 95 % Confidence Interval**	**Upper 95 % Confidence Interval**	**Effect**
mad1	Nascent	-0.11	-0.32	0.18	same
mad1	Mature Nuclear	N/A	N/A	N/A	N/A
mad1	Cytoplasm	0.08	-0.12	0.29	same
rpb1	Nascent	0.08	-0.09	0.24	same
rpb1	Mature Nuclear	0.21	-0.18	0.75	same
rpb1	Cytoplasm	1.21	0.74	1.68	larger

A.5 Comparison of Fano factors between endogenous and exogenous locus and with different coding sequences

We tested if size-corrected Fano factors for *mad2* and *mad3* differ between endogenous and exogenous locus, and after exchanging the coding sequence, using hierarchical bootstrapping. Mean and 95 % confidence intervals from bootstrapping the mean difference are provided. Confidence intervals that exclude 0 are interpreted as a significant difference in Fano factors.

Table A-VI: Comparison of Fano factors for mad2 and mad3 between endogenous and exogenous locus and with different coding sequences

Comparing Fano factors for *mad2* and *mad3* between endogenous and exogenous locus, and at the exogenous locus after changing the coding sequence (see Fig. 5). A positive mean Fano factor difference indicates a larger Fano factor in Genotype 2 than Genotype 1. Change observed in mononucleated cells (as opposed to mononucleated and binucleated cells pooled = all) is indicated in the second Effect column, without listing the mean Fano factor difference and confidence intervals.

**Genotype 1**	**Genotype 2**	**Compart-ment**	**Mean Fano Factor Difference**	**Lower 95 % Confidence Interval**	**Upper 95 % Confidence Interval**	**Effect all**	**Effect mono-nucl.**
mad2 endog	mad2 exog	Nucleus	0.02	-0.07	0.10	same	same
mad2 endog	mad2 exog	Cytoplasm	0.08	-0.05	0.21	same	same
mad2 exog	Pmad2-**rad21** exog	Nucleus	0.12	-0.04	0.29	same	same
mad2 exog	Pmad2-**rad21** exog	Cytoplasm	0.34	0.14	0.55	larger	larger
mad2 exog	Pmad2-**nmt1** exog	Nucleus	0.10	-0.03	0.22	same	same
mad2 exog	Pmad2-**nmt1** exog	Cytoplasm	0.17	0.00	0.35	same	same
mad3 endog	mad3 exog	Nucleus	0.00	-0.15	0.16	same	same
mad3 endog	mad3 exog	Cytoplasm	0.26	0.14	0.38	larger	larger
mad3 exog	Pmad3-**rad21** exog	Nucleus	-0.07	-0.26	0.11	same	same
mad3 exog	Pmad3-**rad21** exog	Cytoplasm	0.39	0.17	0.62	larger	larger
mad3 exog	Pmad3-**nmt1** exog	Nucleus	-0.07	-0.27	0.12	same	same
mad3 exog	Pmad3-**nmt1** exog	Cytoplasm	0.16	-0.04	0.36	same	larger

Section B: Modeling and Inference

B.1 Model specification

Transcription is modelled by the set of (effective) reactions:

$$U_1 \to U_2 \to \cdots \to U_S \to U_1 + N$$

$$N \Rightarrow M_N$$

where *N* is the nascent RNA and M_N is the mature nuclear mRNA. We consider *S* gene states, U_i, where *i* = 1, …, *S*. The free promoter state U_1 denotes the state in which no RNA polymerase (RNAP) is bound to the promoter. Once RNAP binds the free promoter, a closed complex between the two is formed (U_2). Downstream steps (U_3 to U_{S-1}) may include several long-lived intermediate states related to promoter opening and escape (*3–8*). The last state U_S is a promoter-proximal paused state; release from this state leads to the clearing of the promoter for new RNAP binding and the beginning of elongation of the nascent transcript (*9*). This is modelled by the reaction $U_S \to U_1 + N$. After a time delay, elongation and termination finish, leading to mature nuclear mRNA M_N. The rate of switching from $U_i \to U_{i+1}$ is given by k_A, and the rate of initiating nascent mRNA production, $U_S \to U_1 + N$, is given by k_B. The time for the nascent RNA (N) to become mature mRNA (M_N) is considered fixed at some value T, i.e. the reaction $N \Rightarrow M_N$ is a delayed reaction (denoted by the double right arrow). The fixed delay (deterministic elongation and termination) can be derived from a microscopic stochastic model of RNAP movement along the DNA template (*10*), and deterministic elongation has been observed in budding yeast (*11*). Similar models have been proposed (*6*, *12–14*). Note that in this model there is no transcriptionally inactive state in the sense that each state is associated with a particular stage of the transcription process.

Nuclear export is modelled by the reaction

$$M_N \to M_C^1$$

with rate k_C. This process is modelled by a single rate-limiting step. M_C^1 is to be interpreted as the mRNA upon its entrance into the cytoplasm.

Degradation of mRNA in the cytoplasm is modelled by the chain of reactions

$$M_C^1 \to M_C^2 \to \cdots \to M_C^R \to \emptyset$$

Cytoplasmic mRNA degradation in eukaryotes is a complex multi-step process (*15*, *16*) that we simplify to the chain of reactions above. We are not aware of specific data for the number of rate-

limiting steps *R* in the degradation process. In Deneke et al., 2013 (*16*), it was shown that choosing R = 5 can fit data in yeast well, though other values may provide an equally good fit. The rate of switching from $M_C^i \to M_C^{i+1}$ is given by k_D. Note that the measured cytoplasmic mRNA is assumed to be the sum of cytoplasmic mRNA at any stage of its cytoplasmic lifecycle, i.e. $M_C = \sum_{i=1}^{R} M_C^i$.

B.2 General properties of the model

The model is characterized by four rate constants k_A, k_B, k_C *and* k_D, the number of rate-limiting steps in the initiation process *(S)*, and the number of rate-limiting steps in cytoplasmic degradation *(R)*.

The mean time between two successive nascent mRNA production events is given by:

$$T_{init} = \frac{S-1}{k_A} + \frac{1}{k_B}$$

The mean time for nuclear export is:

$$T_{expt} = \frac{1}{k_C}$$

The mean time for cytoplasmic degradation is:

$$T_{degr} = \frac{R}{k_D}$$

The chemical master equation of the model is difficult to solve exactly, even in steady-state conditions. This is not unexpected since exact solutions for the joint distributions of molecule numbers are only known for a handful of special cases (*17*). However, because all reactions are first order, the propensities of the chemical master equation are linear in the molecule numbers. This implies that the means, variances and covariances of all species can be obtained exactly in closed-form using the linear-noise approximation (*17*). The matrix form of this method (*18*) is particularly useful because it makes computations using a computer algebra system such as Mathematica easy for any number of species (which can be arbitrarily large for our model depending on the values of *S* and *R*).

As in the telegraph model of gene expression (*19*), in steady-state conditions the moments of nuclear and cytoplasmic mRNA are functions not of the absolute values of the rate constants but rather of the rate constants normalized by the degradation rate, i.e. k_A / k_D, k_B / k_D, k_C / k_D. Hence, without loss of generality we set k_D = R such that T_{degr} is fixed to unity; in other words, this choice non-dimensionalizes time by dividing it by the mean time it takes for a cytoplasmic mRNA to

decay.

It is straightforward to derive the first-order moments of nuclear and cytoplasmic mRNA numbers per gene copy. These are respectively given by: $\mu_N = \frac{k_A k_B}{k_C(k_A + k_B(S-1))}$ and $\mu_C = \frac{k_A k_B}{k_A + k_B(S-1)}$.

The closed-form solutions for the second-order moments of mRNA numbers were obtained using Mathematica. They are very complex and hence we do not show them here. Instead, we state properties of the stochastic model that follow from an analysis of these solutions:

For all parameters, the Fano factors of nuclear mRNA and of cytoplasmic mRNA are found to be less than or equal to 1. For S = 1, they are exactly equal to 1. As shown in previous publications (*13*, *14*), the crucial ingredient of the model that leads to sub-Poisson noise is the switching of the promoter state upon production of a single nascent transcript, changing it from a state where no new RNAP can bind to a state where this is possible. This captures the observation that a promoter can only bind a new RNAP if there is no other bound RNAP within a short distance from it (*20*, *21*). Mathematically, the switching of the promoter state causes sub-Poisson noise in the transcript numbers because the coefficient of variation of the time between two successive nascent mRNA production events is less than 1, i.e. less than expected from an exponential distribution.

Note that if there was no state switching upon the production of a nascent transcript

$$U_S \to U_1 \to U_2 \to \cdots \to U_S \to U_S + N$$

then the Fano factors of nascent, nuclear and cytoplasmic mRNA would be greater than or equal to 1 (*22*, *23*). In models of this type, the interpretation is that U_1 to U_{S-1} are transcriptional off states while U_S is a transcriptionally active state. Nascent mRNA can be continuously produced from this state and there is no associated promoter clearing.

In steady-state conditions, the dynamics of nascent mRNA elongation and release do not influence the moments of the nuclear and cytoplasmic mRNA in our model. This is because we are assuming that release happens a certain fixed time T after initiation of elongation, and hence the time between two subsequent nascent mRNA production events is the same as the time between two subsequent mature nuclear mRNA production events. In other words, the moments of nuclear and cytoplasmic mRNA in steady-state conditions remain the same if we instead modeled transcription as

$$U_1 \to U_2 \to \cdots \to U_S \to U_1 + M_N$$

where the last reaction has rate constant k_B.

In steady-state conditions, the Fano factor of cytoplasmic mRNA can either be larger or smaller than the Fano factor of nuclear mRNA. From the closed-form solutions it is found that in the limit k_C goes to infinity, the Fano factor of cytoplasmic mRNA is smaller than that of nuclear mRNA. The exact value of k_C when the two Fano factors are equal depends on the values of k_A, k_B, S and R.

The minimum Fano factor of nuclear mRNA is achieved when $k_B = k_A \gg k_C$, in other words when the rates for promoter remodeling and promoter freeing are in the same range and considerably faster than export. The minimum Fano factor of cytoplasmic mRNA is achieved in the limits $k_C \gg k_B = k_A \gg k_D$, in other words when the rates for promoter remodeling and promoter freeing are in the same range and considerably faster than degradation but slower than export. The minimum values possible for a model with S and R each between 1 and 4 are shown in Table B-I and Fig. 7G.

Table B-I: Minimum Fano factor of nuclear and cytoplasmic mRNA

Minimum Fano factors for the general model with S and R rate limiting steps in initiation and decay, respectively. The first number shows the nuclear Fano factor, the second number shows the cytoplasmic Fano factor.

	$R = 1$	$R = 2$	$R = 3$	$R = 4$
$S = 1$	(1.00,1.00)	(1.00,1.00)	(1.00,1.00)	(1.00,1.00)
$S = 2$	(0.750,0.750)	(0.750,0.688)	(0.750,0.656)	(0.750,0.637)
$S = 3$	(0.667,0.667)	(0.667,0.583)	(0.667,0.542)	(0.667,0.516)
$S = 4$	(0.625,0.625)	(0.625,0.531)	(0.625,0.484)	(0.625,0.455)

B.3 Bayesian model selection and parameter inference

While it is immediately clear from Table B-I that models with one rate limiting step for transcription will not be able to explain the sub-Poisson nature of the data, it is an open question which of the models with two or more rate-limiting steps in transcription and one or more rate-limiting steps in degradation best fits the data. To answer this question, we used a simple ABC (Approximate Bayesian Computation) rejection sampler (*24*) to perform Bayesian model selection (*25*).

For each gene and for a given model with certain values of S and R, the ABC algorithm randomly samples parameters k_A, k_B, k_C from a prior distribution. Since we do not have prior knowledge

about the 3 parameters, we simply choose the distribution to be uniform over a certain range. Stochastic simulations of the model with the selected parameters are run using Gillespie's exact algorithm (*26*) until steady-state is achieved. Then a distance function between the moments of the data and the model is evaluated. Note that the number of samples of Gillespie's algorithm equals the number of single-cell measurements for a given gene. This constraint makes sure that the simulation-generated moments have the same variability (due to finite number of samples) as the moments calculated from the data. Those parameters whose distance function is less than a certain threshold are accepted. The mean and standard deviation of the marginal distribution of these accepted parameters (the posterior) provides an estimate of the values of the parameters and their uncertainty. We also calculate the acceptance rate, which is the number of parameter sets that are accepted divided by the total number of parameter sets that were randomly generated by the ABC algorithm. We then select which model best selects the data for a given gene by comparing the acceptance rate of any two models. The Bayes factor of two models A and B is defined as the ratio of the acceptance rate of the two models (*25*). If the Bayes factor is larger than 10, we conclude that there is strong evidence in favor of model A; if the Bayes factor is less than 1/10, we conclude that there is strong evidence in favor of model B (*27*). Otherwise, if the Bayes factor is between 1/10 and 10, we will conclude that there is no strong evidence for model A or model B (there might be weak evidence).

The heart of the ABC algorithm involves accepting only those parameters which satisfy a user-defined criterion. In our case the criterion is that the following distance function is smaller than a preset threshold value:

$$d = \left(\frac{2\mu_C - \mu_C^e}{\mu_C^e}\right)^2 + \left(\frac{FF_C - FF_C^e}{FF_C^e}\right)^2 + \left(\frac{FF_N - FF_N^e}{FF_N^e}\right)^2$$

where μ_C and FF_C are the simulation predictions for the mean and Fano factor (variance divided by the mean) of the cytoplasmic mRNA, M_C; and FF_N is the simulation prediction for the Fano factor of the nuclear mRNA, M_N. The same variables but with an *e* superscript denote the experimental estimates of the mean and the Fano factors for non-transcription site (non-TS) labelled data from mononucleated cells. Note that in this section whenever we refer to the experimental mean of transcript numbers or the Fano factor, we always mean the volume-corrected values (see Methods). This is necessary since the model's predictions (with which the experimental values will be compared) are independent of the cell volume. Note also that the simulation predictions are for a single gene copy. Mononucleated *S. pombe* cells are almost exclusively in G2 phase of the cell cycle and hence have two (independent) gene copies (*28*, *29*).

Therefore, we multiply the model's estimate of the mean by 2 in the expression for the distance function. The Fano factor is unchanged because both the mean and variance are doubled.

Note that the distance function does not have information about the mean of nuclear mRNA but only about its Fano factor. This choice is motivated by a direct comparison of fitting to the data with or without the transcription site label for the two genes, *mad1* and *rpb1*. When the transcription site is not labelled (non-TS labelled), it is uncertain which fraction of the nuclear RNA is nascent RNA and which fraction is mature nuclear RNA. Not making this distinction leads to a considerably different estimate for the mean of nuclear RNA, μ_N, from non-TS labelled data compared to that estimated from TS-labelled data (Table B-II). The relative error is around 200 %. In contrast, estimating the nuclear Fano factor, FF_N, is more robust (relative error less than 20 %). Furthermore, the estimates for both cytoplasmic mean and cytoplasmic Fano factor are similar, regardless of which data is used. We therefore excluded the mean of nuclear RNA, μ_N, from the distance function.

Table B-II: Comparison of the means (μ) and Fano factors of nuclear (N) and cytoplasmic (C) mRNA

Parameters were estimated using both non-TS labelled and TS-labelled data for both genes. The largest discrepancies are observed for μ_N (red).

	μ_N		μ_C		FF_N		FF_C	
	non-TS labelled	TS-labelled	non-TS labelled	TS-labelled	non-TS labelled	TS-labelled	non-TS labelled	TS-labelled
mad1	0.318	0.120	1.74	1.74	0.929	0.917	0.620	0.540
rpb1	3.56	1.15	17.9	15.7	0.826	0.939	0.647	0.560

The only remaining information to be specified for the ABC rejection sampler is the choice of the ranges over which the parameters are randomly sampled and the threshold values (d_c) of the distance function for each gene (Table B-III). We have not included *cdc13* in this analysis because its data is consistent with a Poisson distribution (Section A.4). Theoretically it is known that the distribution of accepted parameters from an ABC rejection sampler will be a good approximation to the true posterior distribution provided the threshold value is sufficiently small (*25*). A threshold value is hence chosen small enough so that repeating with a smaller value does not significantly change the posterior; this value will necessarily be different from one gene to another, particularly given the variable sample sizes. The range of the uniform prior distribution was initially chosen to

be (0,250) for k_A, k_B, k_C. We noticed that in many cases initial runs of the ABC rejection sampler identified parameters satisfying the user-defined criterion only in small regions of this range. Hence to obtain a large number of accepted parameters within a computationally reasonable time (which is needed to obtain a well-defined posterior distribution) it was necessary to limit the range of the prior distributions to a subset of numbers within (0,250) which the initial exploration identified as containing the vast majority of the accepted parameters. We implemented the constraint that, for a given parameter set, the 12 models should be indistinguishable with respect to the mean nuclear and cytoplasmic mRNA. This is achieved by scaling k_A by a factor proportional to $S-1$ (following from the equations for the first-order moments above).

Table B-III: Priors for parameters k_A, k_B, k_C, and thresholds for the distance function (d_c)

Note that $k_D = R$.

Gene	k_A	k_B	k_C	d_c
mad1	$\frac{S-1}{2}(0.5,10.5)$	(0.5,10.5)	(0.5,100.5)	0.003
mad1 − GFP	$\frac{S-1}{2}(0.5,10.5)$	(0.5,40.5)	(20,220)	0.0001
mad2 − GFP	$\frac{S-1}{2}(0.5,35.5)$	(0.5,10.5)	(0.5,20.5)	0.005
mad3 − GFP	$\frac{S-1}{2}(0.5,20.5)$	(0.5,10.5)	(20,220)	0.00025
bub1 − GFP	$\frac{S-1}{2}(0.5,20.5)$	(0.5,75.5)	(20,220)	0.0001
rpb1	$\frac{S-1}{2}(25,75)$	(0.5,30.5)	(0.5,50.5)	0.00075
sep1 − GFP	$\frac{S-1}{2}(0.5,100.5)$	(0.5,5.5)	(0.1,30.1)	0.00075

The Bayes factors were computed for all genes and for all 12 models with $2 \leq S \leq 4$ and $1 \leq R \leq 4$. The results are shown in Tables B-IV-X. For a given gene, the models highlighted in yellow are those which can explain the data, i.e. no other model was found to have an acceptance rate at least 10-times larger; or, in other words, all the 12 Bayes factors for this model are greater than or equal to 1/10. The model selection results are summarized in Table B-XI. We find strong evidence that *mad1*, *mad1*-GFP, and *bub1*-GFP are best described by models with 3 or 4 rate-limiting steps in transcription and 2 – 4 rate-limiting steps in degradation. In contrast, for the rest

of the genes (*mad2*-GFP, *mad3*-GFP, *rpb1*, *sep1*-GFP), most of the 12 models fit the data equally well. We also note that the models with S = 3 and R = 3 or 4, as well as S = 4 and R = 2, are the *only* models that can explain the data from all genes.

Table B-IV: Bayes factors for mad1

The value in the ith row and j^{th} column is the Bayes factor of models i and j. Bayes factors ≥ 10 are shown in blue (model i explains the data better than model j); Bayes factors ≤ 1/10 are shown in red (model j explains the data better than model i); Bayes factors in the range (1/10,10) are shown in black, which is interpreted as the two models being indistinguishable. Those models which explain the data best, i.e. those lacking Bayes factors ≤ 1/10, are shaded in yellow.

mad1	S2_R1	S2_R2	S2_R3	S2_R4	S3_R1	S3_R2	S3_R3	S3_R4	S4_R1	S4_R2	S4_R3	S4_R4
**S2_R1**	1.0E+00	1.0E+00	1.0E+00	2.3E+00	1.3E+00	5.8E+02	4.3E+03	8.1E+03	6.5E+01	9.0E+03	9.5E+03	4.8E+03
**S2_R2**	1.0E+00	1.0E+00	1.0E+00	2.3E+00	1.3E+00	5.8E+02	4.3E+03	8.1E+03	6.5E+01	9.0E+03	9.5E+03	4.8E+03
**S2_R3**	1.0E+00	1.0E+00	1.0E+00	2.3E+00	5.7E-01	5.8E+02	4.3E+03	8.1E+03	6.5E+01	9.0E+03	9.5E+03	4.8E+03
**S2_R4**	4.4E-01	4.4E-01	4.4E-01	1.0E+00	5.7E-01	2.5E+02	1.9E+03	3.5E+03	2.8E+01	3.9E+03	4.2E+03	2.1E+03
**S3_R1**	7.8E-01	7.8E-01	7.8E-01	1.8E+00	1.0E+00	4.5E+02	3.3E+03	6.3E+03	5.0E+01	7.0E+03	7.4E+03	3.8E+03
**S3_R2**	1.7E-03	1.7E-03	1.7E-03	4.0E-03	2.2E-03	1.0E+00	7.4E+00	1.4E+01	1.1E-01	1.6E+01	1.7E+01	8.4E+00
**S3_R3**	2.3E-04	2.3E-04	2.3E-04	5.3E-04	3.0E-04	1.3E-01	1.0E+00	1.9E+00	1.5E-02	2.1E+00	2.2E+00	1.1E+00
**S3_R4**	1.2E-04	1.2E-04	1.2E-04	2.8E-04	1.6E-04	7.1E-02	5.3E-01	1.0E+00	8.0E-03	1.1E+00	1.2E+00	6.0E-01
**S4_R1**	1.5E-02	1.5E-02	1.5E-02	3.5E-02	2.0E-02	8.9E+00	6.6E+01	1.2E+02	1.0E+00	1.4E+02	1.5E+02	7.5E+01
**S4_R2**	1.1E-04	1.1E-04	1.1E-04	2.5E-04	1.4E-04	6.4E-02	4.8E-01	9.0E-01	7.2E-03	1.0E+00	1.1E+00	5.4E-01
**S4_R3**	1.0E-04	1.0E-04	1.0E-04	2.4E-04	1.4E-04	6.0E-02	4.5E-01	8.5E-01	6.8E-03	9.4E-01	1.0E+00	5.1E-01
**S4_R4**	2.1E-04	2.1E-04	2.1E-04	4.7E-04	2.7E-04	1.2E-01	8.9E-01	1.7E+00	1.3E-02	1.9E+00	2.0E+00	1.0E+00

Table B-V: Bayes factors for mad1-GFP

mad1-GFP	S2_R1	S2_R2	S2_R3	S2_R4	S3_R1	S3_R2	S3_R3	S3_R4	S4_R1	S4_R2	S4_R3	S4_R4
**S2_R1**	1.0E+00	1.0E+00	1.9E+01	4.7E+01	1.3E+01	6.4E+02	9.9E+02	8.8E+02	4.0E+02	2.4E+02	7.4E+01	6.6E+01
**S2_R2**	1.0E+00	1.0E+00	1.9E+01	4.7E+01	1.3E+01	6.4E+02	9.9E+02	8.8E+02	4.0E+02	2.4E+02	7.4E+01	6.6E+01
**S2_R3**	5.1E-02	5.1E-02	1.0E+00	2.4E+00	6.6E-01	3.3E+01	5.1E+01	4.5E+01	2.0E+01	1.2E+01	3.8E+00	3.4E+00
**S2_R4**	2.1E-02	2.1E-02	4.2E-01	1.0E+00	2.7E-01	1.4E+01	2.1E+01	1.9E+01	8.4E+00	5.0E+00	1.6E+00	1.4E+00
**S3_R1**	7.8E-02	7.8E-02	1.5E+00	3.6E+00	1.0E+00	5.0E+01	7.7E+01	6.8E+01	3.1E+01	1.8E+01	5.8E+00	5.1E+00
**S3_R2**	1.6E-03	1.6E-03	3.0E-02	7.3E-02	2.0E-02	1.0E+00	1.5E+00	1.4E+00	6.1E-01	3.7E-01	1.2E-01	1.0E-01
**S3_R3**	1.0E-03	1.0E-03	2.0E-02	4.7E-02	1.3E-02	6.5E-01	1.0E+00	8.9E-01	4.0E-01	2.4E-01	7.5E-02	6.7E-02
**S3_R4**	1.1E-03	1.1E-03	2.2E-02	5.3E-02	1.5E-02	7.3E-01	1.1E+00	1.0E+00	4.5E-01	2.7E-01	8.4E-02	7.5E-02
**S4_R1**	2.5E-03	2.5E-03	4.9E-02	1.2E-01	3.3E-02	1.6E+00	2.5E+00	2.2E+00	1.0E+00	5.9E-01	1.9E-01	1.7E-01
**S4_R2**	4.3E-03	4.3E-03	8.3E-02	2.0E-01	5.5E-02	2.7E+00	4.2E+00	3.7E+00	1.7E+00	1.0E+00	3.2E-01	2.8E-01
**S4_R3**	1.3E-02	1.3E-02	2.6E-01	6.3E-01	1.7E-01	8.7E+00	1.3E+01	1.2E+01	5.3E+00	3.2E+00	1.0E+00	8.9E-01
**S4_R4**	1.5E-02	1.5E-02	2.9E-01	7.1E-01	1.9E-01	9.7E+00	1.5E+01	1.3E+01	6.0E+00	3.6E+00	1.1E+00	1.0E+00

Table B-VI: Bayes factors for mad2-GFP

mad2-GFP	S2_R1	S2_R2	S2_R3	S2_R4	S3_R1	S3_R2	S3_R3	S3_R4	S4_R1	S4_R2	S4_R3	S4_R4
**S2_R1**	1.0E+00	8.9E-01	7.4E-01	6.6E-01	6.0E-01	4.2E-01	3.5E-01	3.4E-01	5.3E-01	4.3E-01	3.8E-01	3.5E-01
**S2_R2**	1.1E+00	1.0E+00	8.4E-01	7.4E-01	6.8E-01	4.7E-01	3.9E-01	3.8E-01	6.0E-01	4.8E-01	4.2E-01	3.9E-01
**S2_R3**	1.3E+00	1.2E+00	1.0E+00	8.8E-01	8.1E-01	5.7E-01	4.7E-01	4.5E-01	7.1E-01	5.8E-01	5.1E-01	4.6E-01
**S2_R4**	1.5E+00	1.4E+00	1.1E+00	1.0E+00	9.2E-01	6.4E-01	5.3E-01	5.2E-01	8.1E-01	6.6E-01	5.8E-01	5.3E-01
**S3_R1**	1.7E+00	1.5E+00	1.2E+00	1.1E+00	1.0E+00	7.0E-01	5.8E-01	5.6E-01	8.8E-01	7.2E-01	6.3E-01	5.8E-01
**S3_R2**	2.4E+00	2.1E+00	1.8E+00	1.6E+00	1.4E+00	1.0E+00	8.3E-01	8.0E-01	1.3E+00	1.0E+00	9.0E-01	8.2E-01
**S3_R3**	2.9E+00	2.5E+00	2.1E+00	1.9E+00	1.7E+00	1.2E+00	1.0E+00	9.7E-01	1.5E+00	1.2E+00	1.1E+00	9.9E-01
**S3_R4**	3.0E+00	2.6E+00	2.2E+00	1.9E+00	1.8E+00	1.2E+00	1.0E+00	1.0E+00	1.6E+00	1.3E+00	1.1E+00	1.0E+00
**S4_R1**	1.9E+00	1.7E+00	1.4E+00	1.2E+00	1.1E+00	7.9E-01	6.6E-01	6.4E-01	1.0E+00	8.1E-01	7.1E-01	6.5E-01
**S4_R2**	2.3E+00	2.1E+00	1.7E+00	1.5E+00	1.4E+00	9.8E-01	8.1E-01	7.9E-01	1.2E+00	1.0E+00	8.8E-01	8.0E-01
**S4_R3**	2.7E+00	2.4E+00	2.0E+00	1.7E+00	1.6E+00	1.1E+00	9.2E-01	9.0E-01	1.4E+00	1.1E+00	1.0E+00	9.2E-01
**S4_R4**	2.9E+00	2.6E+00	2.2E+00	1.9E+00	1.7E+00	1.2E+00	1.0E+00	9.8E-01	1.5E+00	1.2E+00	1.1E+00	1.0E+00

Table B-VII: Bayes factors for mad3-GFP

mad3-GFP	S2_R1	S2_R2	S2_R3	S2_R4	S3_R1	S3_R2	S3_R3	S3_R4	S4_R1	S4_R2	S4_R3	S4_R4
**S2_R1**	1.0E+00	6.1E+01	6.5E+01	5.7E+01	8.3E+01	2.0E+01	2.1E+01	2.1E+01	2.7E+01	2.0E+01	2.0E+01	1.9E+01
**S2_R2**	1.6E-02	1.0E+00	1.1E+00	9.4E-01	1.4E+00	3.3E-01	3.4E-01	3.5E-01	4.4E-01	3.3E-01	3.3E-01	3.2E-01
**S2_R3**	1.5E-02	9.3E-01	1.0E+00	8.8E-01	1.3E+00	3.1E-01	3.2E-01	3.2E-01	4.1E-01	3.0E-01	3.1E-01	2.9E-01
**S2_R4**	1.7E-02	1.1E+00	1.1E+00	1.0E+00	1.5E+00	3.5E-01	3.6E-01	3.7E-01	4.7E-01	3.5E-01	3.5E-01	3.4E-01
**S3_R1**	1.2E-02	7.3E-01	7.9E-01	6.9E-01	1.0E+00	2.4E-01	2.5E-01	2.5E-01	3.2E-01	2.4E-01	2.4E-01	2.3E-01
**S3_R2**	5.0E-02	3.0E+00	3.3E+00	2.9E+00	4.2E+00	1.0E+00	1.0E+00	1.1E+00	1.3E+00	1.0E+00	1.0E+00	9.6E-01
**S3_R3**	4.8E-02	2.9E+00	3.1E+00	2.8E+00	4.0E+00	9.6E-01	1.0E+00	1.0E+00	1.3E+00	9.6E-01	9.7E-01	9.3E-01
**S3_R4**	4.7E-02	2.9E+00	3.1E+00	2.7E+00	3.9E+00	9.5E-01	9.9E-01	1.0E+00	1.3E+00	9.5E-01	9.6E-01	9.1E-01
**S4_R1**	3.7E-02	2.3E+00	2.4E+00	2.1E+00	3.1E+00	7.4E-01	7.7E-01	7.8E-01	1.0E+00	7.4E-01	7.5E-01	7.2E-01
**S4_R2**	5.0E-02	3.1E+00	3.3E+00	2.9E+00	4.2E+00	1.0E+00	1.0E+00	1.1E+00	1.4E+00	1.0E+00	1.0E+00	9.7E-01
**S4_R3**	4.9E-02	3.0E+00	3.2E+00	2.8E+00	4.1E+00	9.9E-01	1.0E+00	1.0E+00	1.3E+00	9.8E-01	1.0E+00	9.5E-01
**S4_R4**	5.2E-02	3.2E+00	3.4E+00	3.0E+00	4.3E+00	1.0E+00	1.1E+00	1.1E+00	1.4E+00	1.0E+00	1.1E+00	1.0E+00

Table B-VIII: Bayes factors for bub1-GFP

bub1-GFP	S2_R1	S2_R2	S2_R3	S2_R4	S3_R1	S3_R2	S3_R3	S3_R4	S4_R1	S4_R2	S4_R3	S4_R4
**S2_R1**	1.0E+00	2.8E+00	1.3E+01	5.4E+01	1.5E+01	8.8E+02	1.5E+03	1.5E+03	3.9E+02	1.0E+03	2.1E+02	1.6E+02
**S2_R2**	3.6E-01	1.0E+00	4.7E+00	1.9E+01	5.4E+00	3.1E+02	5.2E+02	5.2E+02	1.4E+02	3.6E+02	7.3E+01	5.6E+01
**S2_R3**	7.7E-02	2.1E-01	1.0E+00	4.1E+00	1.2E+00	6.8E+01	1.1E+02	1.1E+02	3.0E+01	7.8E+01	1.6E+01	1.2E+01
**S2_R4**	1.9E-02	5.2E-02	2.4E-01	1.0E+00	2.8E-01	1.6E+01	2.7E+01	2.7E+01	7.2E+00	1.9E+01	3.8E+00	3.0E+00
**S3_R1**	6.6E-02	1.9E-01	8.6E-01	3.5E+00	1.0E+00	5.8E+01	9.6E+01	9.7E+01	2.6E+01	6.7E+01	1.4E+01	1.0E+01
**S3_R2**	1.1E-03	3.2E-03	1.5E-02	6.1E-02	1.7E-02	1.0E+00	1.7E+00	1.7E+00	4.4E-01	1.1E+00	2.3E-01	1.8E-01
**S3_R3**	6.9E-04	1.9E-03	9.0E-03	3.7E-02	1.0E-02	6.1E-01	1.0E+00	1.0E+00	2.7E-01	7.0E-01	1.4E-01	1.1E-01
**S3_R4**	6.8E-04	1.9E-03	6.5E-01	3.7E-02	1.0E-02	6.0E-01	1.0E+00	1.0E+00	2.6E-01	6.9E-01	1.4E-01	1.1E-01
**S4_R1**	2.6E-03	7.2E-03	3.4E-02	1.4E-01	3.9E-02	2.3E+00	3.8E+00	3.8E+00	1.0E+00	2.6E+00	5.3E-01	4.1E-01
**S4_R2**	9.9E-04	2.8E-03	1.3E-02	5.3E-02	1.5E-02	8.7E-01	1.4E+00	1.4E+00	3.8E-01	1.0E+00	2.0E-01	1.6E-01
**S4_R3**	4.9E-03	1.4E-02	6.4E-02	2.6E-01	7.4E-02	4.3E+00	7.1E+00	7.1E+00	1.9E+00	5.0E+00	1.0E+00	7.7E-01
**S4_R4**	6.3E-03	1.8E-02	8.2E-02	3.4E-01	9.6E-02	5.6E+00	7.0E+00	9.2E+00	2.4E+00	6.4E+00	1.3E+00	1.0E+00

Table B-IX: Bayes factors for rpb1

rpb1	S2_R1	S2_R2	S2_R3	S2_R4	S3_R1	S3_R2	S3_R3	S3_R4	S4_R1	S4_R2	S4_R3	S4_R4
**S2_R1**	1.0E+00	1.5E+02	9.5E+02	1.9E+03	8.4E+02	1.6E+03	1.1E+03	9.3E+02	1.9E+03	1.1E+03	1.0E+03	9.8E+02
**S2_R2**	6.6E-03	1.0E+00	6.3E+00	1.2E+01	5.5E+00	1.0E+01	7.3E+00	6.2E+00	1.3E+01	7.5E+00	6.8E+00	6.5E+00
**S2_R3**	1.1E-03	1.6E-01	1.0E+00	2.0E+00	8.8E-01	1.7E+00	1.2E+00	9.8E-01	2.0E+00	1.2E+00	1.1E+00	1.0E+00
**S2_R4**	5.3E-04	8.0E-02	5.0E-01	1.0E+00	4.5E-01	8.4E-01	5.9E-01	5.0E-01	1.0E+00	6.0E-01	5.4E-01	5.2E-01
**S3_R1**	1.2E-03	1.8E-01	1.1E+00	2.2E+00	1.0E+00	1.9E+00	1.3E+00	1.1E+00	2.3E+00	1.3E+00	1.2E+00	1.2E+00
**S3_R2**	6.3E-04	9.6E-02	6.0E-01	1.2E+00	5.3E-01	1.0E+00	7.1E-01	5.9E-01	1.2E+00	7.2E-01	6.5E-01	6.2E-01
**S3_R3**	9.0E-04	1.4E-01	8.5E-01	1.7E+00	7.5E-01	1.4E+00	1.0E+00	8.4E-01	1.7E+00	1.0E+00	9.2E-01	8.8E-01
**S3_R4**	1.1E-03	1.6E-01	1.0E+00	2.0E+00	9.0E-01	1.7E+00	1.2E+00	1.0E+00	2.1E+00	1.2E+00	1.1E+00	1.1E+00
**S4_R1**	5.2E-04	7.9E-02	4.9E-01	9.8E-01	4.4E-01	8.2E-01	5.8E-01	4.8E-01	1.0E+00	5.9E-01	5.3E-01	5.1E-01
**S4_R2**	8.8E-04	1.3E-01	8.4E-01	1.7E+00	7.4E-01	1.4E+00	9.8E-01	8.2E-01	1.7E+00	1.0E+00	9.0E-01	8.7E-01
**S4_R3**	9.8E-04	1.5E-01	9.3E-01	1.8E+00	8.2E-01	1.5E+00	1.1E+00	9.1E-01	1.9E+00	1.1E+00	1.0E+00	9.6E-01
**S4_R4**	1.0E-03	1.5E-01	9.7E-01	1.9E+00	8.6E-01	1.6E+00	1.1E+00	9.5E-01	2.0E+00	1.2E+00	1.0E+00	1.0E+00

Table B-X: Bayes factors for sep1-GFP

sep1-GFP	S2_R1	S2_R2	S2_R3	S2_R4	S3_R1	S3_R2	S3_R3	S3_R4	S4_R1	S4_R2	S4_R3	S4_R4
**S2_R1**	1.0E+00	8.0E-01	7.4E-01	7.1E-01	9.9E-01	8.4E-01	7.7E-01	7.3E-01	1.0E+00	8.6E-01	8.1E-01	7.5E-01
**S2_R2**	1.2E+00	1.0E+00	9.2E-01	8.8E-01	1.2E+00	1.1E+00	9.6E-01	9.1E-01	1.3E+00	1.1E+00	1.0E+00	9.4E-01
**S2_R3**	1.4E+00	1.1E+00	1.0E+00	9.6E-01	1.3E+00	1.1E+00	1.0E+00	9.9E-01	1.4E+00	1.2E+00	1.1E+00	1.0E+00
**S2_R4**	1.4E+00	1.1E+00	1.0E+00	1.0E+00	1.4E+00	1.2E+00	1.1E+00	1.0E+00	1.4E+00	1.2E+00	1.1E+00	1.1E+00
**S3_R1**	1.0E+00	8.1E-01	7.4E-01	7.1E-01	1.0E+00	8.5E-01	7.8E-01	7.3E-01	1.0E+00	8.7E-01	8.2E-01	7.6E-01
**S3_R2**	1.2E+00	9.5E-01	8.8E-01	8.4E-01	1.2E+00	1.0E+00	9.1E-01	8.6E-01	1.2E+00	1.0E+00	9.6E-01	9.0E-01
**S3_R3**	1.3E+00	1.0E+00	9.6E-01	9.2E-01	1.3E+00	1.1E+00	1.0E+00	9.5E-01	1.3E+00	1.1E+00	1.1E+00	9.8E-01
**S3_R4**	1.4E+00	1.1E+00	1.0E+00	9.7E-01	1.4E+00	1.2E+00	1.1E+00	1.0E+00	1.4E+00	1.2E+00	1.1E+00	1.0E+00
**S4_R1**	9.9E-01	7.9E-01	7.3E-01	7.0E-01	9.8E-01	8.3E-01	7.6E-01	7.2E-01	1.0E+00	8.5E-01	8.0E-01	7.5E-01
**S4_R2**	1.2E+00	9.3E-01	8.6E-01	8.2E-01	1.1E+00	9.8E-01	8.9E-01	8.4E-01	1.2E+00	1.0E+00	9.4E-01	8.7E-01
**S4_R3**	1.2E+00	9.9E-01	9.1E-01	8.7E-01	1.2E+00	1.0E+00	9.5E-01	9.0E-01	1.2E+00	1.1E+00	1.0E+00	9.3E-01
**S4_R4**	1.3E+00	1.1E+00	9.8E-01	9.4E-01	1.3E+00	1.1E+00	1.0E+00	9.6E-01	1.3E+00	1.1E+00	1.1E+00	1.0E+00

Table B-XI: Summary of Bayesian model selection results

The symbol X denotes a model which can explain the data for a particular gene, i.e. all the 12 Bayes factors for this model are greater than or equal to 1/10. Those models that explain data from all genes are shaded in yellow.

	**S2_R1**	**S2_R2**	**S2_R3**	**S2_R4**	**S3_R1**	**S3_R2**	**S3_R3**	**S3_R4**	**S4_R1**	**S4_R2**	**S4_R3**	**S4_R4**
mad1							X	X		X	X	X
mad1 – GFP						X	X	X	X	X		
mad2 – GFP	X	X	X	X	X	X	X	X	X	X	X	X
mad3 – GFP		X	X	X	X	X	X	X	X	X	X	X
bub1 – GFP						X	X	X	X	X	X	X
rpb1			X	X	X	X	X	X	X	X	X	X
sep1 – GFP	X	X	X	X	X	X	X	X	X	X	X	X

Furthermore, from the posterior distributions we can estimate the value of the parameters and quantify their uncertainty. For this purpose, we used the model S = R = 3 since it is one of the few models which can explain the data from all genes. In Table B-XII we show the estimates of the three parameters and their uncertainty (mean and standard deviation of their posterior distributions), as well as two biologically meaningful functions of these parameters:

- T_{init}/T_{degr}, the ratio of the mean time between two successive transcription events and the mean time between two successive cytoplasmic degradation events, and
- T_{expt}/T_{init}, the ratio of the mean time for nuclear export and the mean time between two successive transcription events.

Computing percentage error as standard deviation divided by the mean, the percentage error is in the range 10–36 % for k_A, 4–69 % for k_B, 21–66 % for k_C, 1.4–3.3 % for T_{init}/T_{degr}, and 26–186 % for T_{expt}/T_{init}. Hence, the most clearly identifiable parameter is T_{init}/T_{degr}. The underlying reason is that this parameter is equal to the model's prediction of the inverse of the cytoplasmic mean which has been directly measured. The large errors in T_{expt}/T_{init} are to be expected because this quantity is equal to the model's prediction of the nuclear mean which we do not use in our distance function since the estimated mean from experimental data without transcription site label is significantly different from that estimated from data with transcription site label. Nevertheless, the mean nuclear mRNA per gene copy predicted for *mad1* and *rpb1* from non-TS labelled data are in good agreement with those measured in the TS-labelled data (Table B-XII),

which is remarkable given that the TS-labelled data was not used to estimate parameters.

Note that the posterior distributions are not uniform (as the priors), but peaked (Figure B-I), implying that the inference procedure was successful at finding optimal parameter values for each gene. The identified parameters varied across genes (Figure B-I,B-II).

Table B-XII: Estimation of parameters from non-TS labelled data

Estimation uses the S = R = 3 model with k_D=R. In brackets we show the estimated uncertainty; this is computed using the standard deviation of the accepted ABC sample estimates. T_{expt}/T_{init} is equal to the model's prediction of the nuclear mean mature nuclear mRNA number. In the last column, for comparison, we show the mean mature nuclear mRNA number per gene copy from TS-labelled data (Fig. 7D). Note that the choice k_D=R implies that k_A, k_B, k_C have units of inverse time where one time unit equals the mean time for one cytoplasmic mRNA molecule to decay.

**Gene**	$\boldsymbol{k_A}$	$\boldsymbol{k_B}$	$\boldsymbol{k_C}$	$\boldsymbol{T_{init}/T_{degr}}$	$\boldsymbol{T_{expt}/T_{init}}$	mean mature nuclear mRNA per gene copy
mad1	2.54 (0.504)	3.64 (1.79)	20.0 (9.08)	1.15 (0.0366)	0.0533 (0.0248)	0.064
mad1 − GFP	3.49 (1.24)	20.4 (9.92)	111 (37.9)	0.7 (0.0106)	0.0145 (0.00559)	N/A
mad2 − GFP	14.8 (3.46)	1.84 (0.125)	4.33 (2.33)	0.689 (0.0230)	0.453 (0.271)	N/A
mad3 − GFP	13.3 (1.39)	2.42 (0.0997)	90.4 (31.5)	0.567 (0.00804)	0.0220 (0.00851)	N/A
bub1 − GFP	5.62 (1.80)	26.9 (18.5)	45.5 (9.77)	0.455 (0.00732)	0.0510 (0.0133)	N/A
rpb1	49.2 (5.82)	14.3 (1.07)	27.0 (9.35)	0.112 (0.00157)	0.382 (0.164)	0.604
sep1 − GFP	53.7 (19.1)	3.08 (0.179)	10.0 (6.61)	0.368 (0.00983)	0.636 (1.18)	N/A

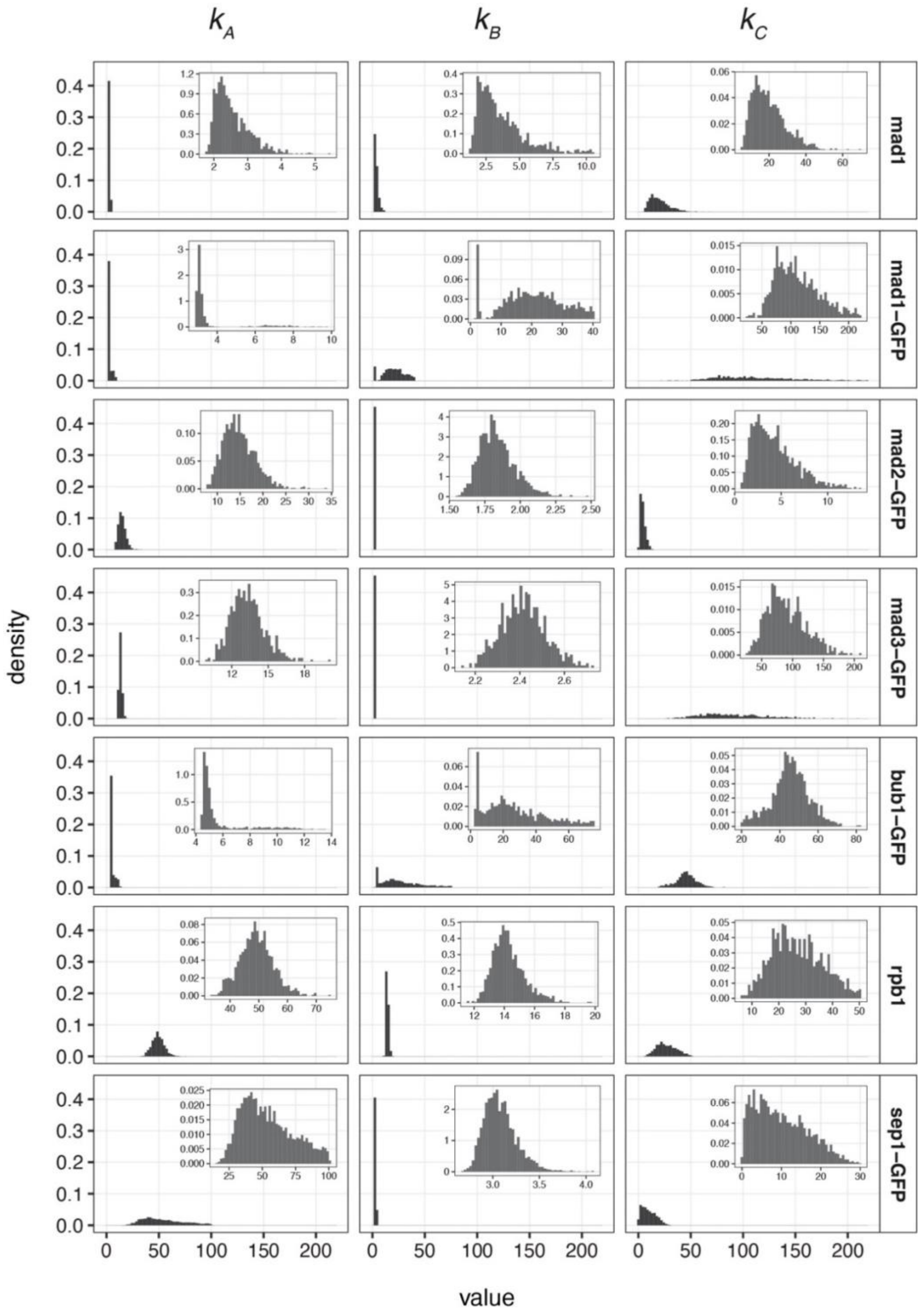

Figure B-I: Posterior distributions of k_A, k_B, and k_C

Posterior distributions were obtained using an ABC rejection sampler with the model with S = R = 3.

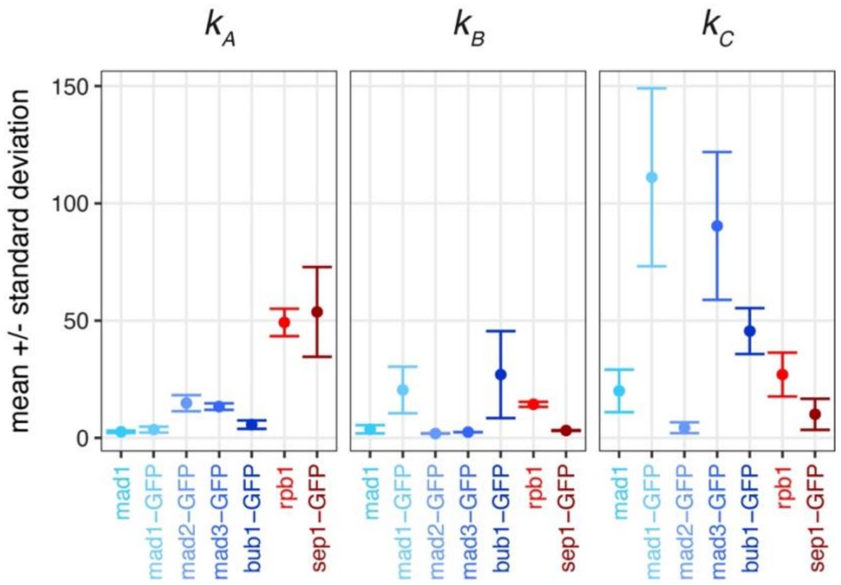

Figure B-II: Overview on estimates of k_A, k_B, and k_C from the posterior distributions

Same data as in Figure B-I. Mean +/- standard deviation is shown.

Table B-XIII: Nuclear export rate k_C above which the Fano factor of cytoplasmic mRNA becomes smaller than the Fano factor of nuclear mRNA

These are computed from the analytical expression of the model assuming $k_A = k_B = 10$.

	$\boldsymbol{R = 1}$	$\boldsymbol{R = 2}$	$\boldsymbol{R = 3}$	$\boldsymbol{R = 4}$
$\mathbf{S = 2}$	4.58	2.60	2.14	1.94
$\mathbf{S = 3}$	3.87	2.38	2.01	1.84
$\mathbf{S = 4}$	3.39	2.21	1.89	1.75

B.4 Testing the ansatz of Poisson fluctuations

We considered the possibility that—while the measurements indicate sub-Poissonian mRNA distributions—they may instead result from a Poisson process and appear sub-Poissonian due to finite sample sizes. Specifically, if *n* Poisson-distributed random variables are generated (mimicking the mRNA number produced with a single rate-limiting step and measured in *n* cells), and the Fano factor (variance / mean) is calculated for this population of cells, then the Fano factor will vary around 1 and has a 50 % chance of being less than 1. In fact, it is known that the sampling distribution of the Fano factor is approximately a gamma distribution with shape

parameter $(n-1)/2$ and scale parameter $2/(n-1)$ (*30*). Hence if the data from *n* cells were generated by a Poisson process, with probability 0.95 the computed Fano factor would lie in the interval $[\lambda_1, \lambda_2]$ where $\int_0^{\lambda_1} \Gamma(x; (n-1)/2, 2/(n-1))dx = 0.025$ and $\int_0^{\lambda_2} \Gamma(x; (n-1)/2, 2/(n-1))dx = 0.975$. In Table B-XIV we show the computation of the 95 % confidence interval for the Fano factor for all genes.

Table B-XIV: Testing if the measured Fano factor could be due to finite sampling of Poisson noise

For each gene we compute the confidence interval (CI) for its Fano factor given the number of cells and assuming mRNA fluctuations are Poissonian. Fano factor values in red indicate those which fall within the confidence intervals.

Gene	Number of cells	Nuclear Fano factor	Cytoplasmic Fano factor	95 % CI for Fano factor
**mad1**	1382	0.929	0.620	[0.927,1.08]
**mad1 − GFP**	2155	0.983	0.657	[0.941,1.06]
**mad2 − GFP**	3064	0.845	0.824	[0.951,1.05]
**mad3 − GFP**	2661	0.974	0.725	[0.947,1.05]
**bub1 − GFP**	1142	0.951	0.621	[0.920,1.08]
**sep1 − GFP**	589	0.865	0.880	[0.889,1.11]
**rpb1**	1425	0.826	0.647	[0.928,1.07]
**cdc13**	1144	0.929	0.997	[0.920,1.08]

Note that *cdc13* is the only gene whose nuclear and cytoplasmic Fano factors both fall within the confidence intervals (also see Fig. A-I). On this basis, we cannot rule out that this gene has Poissonian expression characteristics, and hence we have not used data from this gene when fitting to the model. We note that for some genes their nuclear Fano factor falls within the confidence intervals whereas their cytoplasmic Fano factor does not (Table B-XIV). This might be expected due to the relationship between nuclear and cytoplasmic Fano factors when the nuclear export rate is high (Fig. 7H).

Tables S1 – S4

Table S1: *S. pombe* strains

Strain Number	Mating type	Genotype
JY001	h-	
JY002	h+	
JY265	h-	leu1
JY743	h-	leu1 ura4-D18
SU161	h-	leu1<<343nt-bub1+-ymeGFP-427nt
SU162	h-	leu1<<82nt-bub1+-ymeGFP-427nt
SU163	h-	leu1<<110nt-mad1+-ymeGFP-164nt
SU165	h-	leu1<<13nt-mad1+-ymeGFP-164nt
SU167	h+	leu1<<49nt-mad2+-ymeGFP-521nt
SU169	h-	leu1<<460nt-mad3+-ymeGFP-279nt
SU170	h-	leu1<<286nt-mad3+-ymeGFP-279nt
SU171	h+	wis1+<<86nt-mad1+-ymEGFP-164nt
SU172	h+	wis1+<<48nt-mad1+-ymEGFP-164nt
SU173	h+	wis1+<<20nt-mad1+-ymEGFP-164nt
SU174	h+	wis1+<<13nt-mad1+-ymEGFP-164nt
SU184	h-	wis1+<<286nt-mad3+-ymEGFP-279nt
SU185	h+	wis1+<<130nt-bub1+-ymEGFP-427nt
SU186	h+	mad3Δ::ymEGFP
SU186'	h+	mad3Δ::ymEGFP
SU189	h+	wis1+<<170nt-bub1+-ymEGFP-427nt
SU190	h-	rad21+-ymEGFP<<kanR
SU192	h+	wis1+<<392nt-mad3+-ymEGFP-279nt
SU193	h+	wis1+<<460nt-mad3+-ymEGFP-279nt
SU194	h+	wis1+<<332nt-mad3+-ymEGFP-279nt
SU194'	h+	wis1+<<332nt-mad3+-ymEGFP-279nt
SU216	h-	leu1 ura4-D18 mad2+-ymEGFP
SU228	h+	bub1+-ymEGFP
SU229	h-	bub1+-ymEGFP
SU229'	h-	bub1+-ymEGFP
SU230	h+	bub1Δ::ymEGFP
SU231	h-	bub1Δ::ymEGFP
SU499	h-	leu1 ura4-D18 mad1+-ymEGFP
SU804	h-	leu1 ura4-D18 mad3+-ymEGFP
SW130	h+	mad2+-ymEGFP
SW130'	h+	mad2+-ymEGFP
SW132	h+	mad3+-ymEGFP
SW132'	h+	mad3+-ymEGFP
SW139'	h-	mad2Δ::ymEGFP
SW140'	h+	mad2Δ::ymEGFP
SW176	h+	mad1+-ymEGFP mad3+-ymEGFP
SW201	h-	mad1Δ::ymEGFP
SW203	h-	mad1Δ::ymEGFP
SW204	h+	mad1Δ::ymEGFP
SW205	h+	mad1+-ymEGFP
SW206	h+	mad1+-ymEGFP
SW222	h+	wis1+<<13nt-mad2+-ymEGFP-521nt
SW224	h+	wis1+<<49nt-mad2+-ymEGFP-521nt
SW225	h+	wis1+<<348nt-mad2+-ymEGFP-521nt
SW226	h+	wis1+<<58nt-mad2+-ymEGFP-521nt
SW226'	h+	wis1+<<58nt-mad2+-ymEGFP-521nt
SW228	h+	wis1+<<780nt-mad1+-ymEGFP-164nt
SW229	h+	wis1+<<343nt-bub1+-ymEGFP-427nt
SW231	h+	wis1+<<82nt-bub1+-ymEGFP-427nt
SW232	h+	wis1+<<324nt-bub1+-ymEGFP-427nt
SW235	h-	leu1+<<pDUAL-Pmad1-mad1+-ymEGFP
SW235'	h-	leu1+<<pDUAL-Pmad1-mad1+-ymEGFP
SW241	h-	sep1+-ymEGFP<<hphNT1
SW241'	h-	sep1+-ymEGFP<<hphNT1
SW242	h-	SPAC2H10.01+-ymEGFP<<hphNT1
SW243	h-	SPAC27D7.09c+-ymEGFP<<hphNT1
SW244	h-	leu1+<<pDUAL-Pbub1L-bub1+-ymEGFP
SW244'	h-	leu1+<<pDUAL-Pbub1L-bub1+-ymEGFP
SW245	h-	leu1+<<pDUAL-Pbub1Lmut2-bub1+-ymEGFP
SW245'	h-	leu1+<<pDUAL-Pbub1Lmut2-bub1+-ymEGFP
SW249	h+	GFP-mad1+
SW253	h+	leu1 his7+<<GFP-lacI-NLS but2+<<LEU2+<<lacO<<trz2+
SW254	h+	leu1 his7+<<GFP-lacI-NLS hrd3+<<LEU2+<<lacO<<toa1+
SW509	h-	leu1<<13nt-mad2+-ymEGFP-521nt
SW510	h-	leu1<<348nt-mad2+-ymEGFP-521nt
SW511	h-	leu1<<231nt-mad2+-ymEGFP-521nt
SW513	h+	wis1+<<110nt-mad1+-ymEGFP-164nt
SW514	h-	leu1<<58nt-mad2+-ymEGFP-521nt
SW642	h+	mad1+-ymEGFP
SW702	h+	wis1+<<58nt-Pmad2-rad21+-ymEGFP-521nt
SW703	h+	wis1+<<58nt-Pmad2-nmt1+-ymEGFP-521nt
SW704	h-	leu1+<<pDUAL-Pmad1mut1-mad1+-ymEGFP
SW704'	h-	leu1+<<pDUAL-Pmad1mut1-mad1+-ymEGFP
SW705	h-	leu1+<<pDUAL-Pmad1mut2-mad1+-ymEGFP
SW705'	h-	leu1+<<pDUAL-Pmad1mut2-mad1+-ymEGFP
SW712	h+	wis1+<332nt-Pmad3-nmt1+-ymEGFP-279nt
SW714	h+	wis1+<332nt-Pmad3-rad21+-ymEGFP-279nt

Table S2: sgRNA targeting sequences

Gene	Targeting sequence
mad2	ATTGGGTAGACAGTGACCCT
mad2	TATTCTTCATTAAGTTAGCA
mad2	TGTTGTTCTATTATCACTTT
mad3	GCAATTTACTCACCGTTGGT
wis1 intergenic	GTATGTGGCATACGCAGCCG
leu1 intergenic	GTAAGTACACAGCGACAACT
hph	TGCTCCATACAAGCCAACCA

Table S3: FISH probes

ymEGFP	mad1	rpb1	cdc13
cagtgaataattcttcacctt	gaacggatccctaggagaatc	cgtaaggggacagaagaaggt	aactcgtcaagatgctggtt
tcaaccaaaattgggacaaca	aatctaggcaactgtgaacgc	cggggacaagattccaaattg	taggggaaaagtcctgttct
gaccattaacatcaccatcta	atttggtttcttaacgcttgt	cgcaacgctcattgaacgaat	tctagctctagacagtgact
ccttcaccggagacagaaaat	gcagaattaacagaaggcttc	ttcatccatggtctcaggaaa	ggcaagagcttttgagactt
gtcaatttaccgtaagtagca	agctagtttgggattttttgt	ggtttgacacttgaattgtcg	atagcctttgaccgaatcta
atggaactggcaatttaccag	ccttccgctttaaatcatttt	aaaatgaccaggacaatccgc	aacgtgaaggggtagtacgg
aaagtagtgactaaggttggc	actcaatttgttcacgctcaa	aactggctttgcaagttcaat	acgaattctgtcggggattg
tgttgtttcatatgatctggg	ttcttctgcaagttttctttg	ttttgcttaggaaaccgatgt	taggtgctggcgagttaaac
ctggcatggcagacttgaaaa	gctgtaacgaattcttctgtt	cgcaattccaacaaacgcatt	tttttgcacgggcaatatgg
gttctttcttgaacataacct	agttgcttttcaactagagtt	gatcacgataacgttgtgtgt	gaagacacagtggctttctt
agttaccgtcatctttgaaaa	gataagaagtagactgctcct	cagacattccaaactgcattt	caacgcatgacgcttcttag
ttgacttcagctctggtcttg	ccttttcatttcttacttctt	gtatcgcaaaccatttttgtc	agtgtgtctgacatttgtgt
taactaaggtatcaccttcaa	ttgcatcaagtagttcatgga	aaattatctgagcctgcagat	cacgacgggtactgacagaa
ataccttttaattcgattcta	ttcaactctgcaatctctttc	tattggcggagggattactaa	tcatctgttgcagggattat
cctaaaatgttaccatcttct	gatcattcttttctatctggg	tctttacgtattgtaggttgg	aaacagaaggttggcgacgc
gagagttatagttgtattcca	acttcatgattcttttcactt	caagaaccccataatctcagg	ttgaggtaacgaggggactg
gtcagccatgatgtaaacatt	tgcaaagcttgatttgagacc	aatctgattcatctttaccgc	gttgcttgatccttatggaa
actttgataccattcttttgt	gtttgtaagattggtatcctt	cgacagaaggcgtttttctgg	cgcgttcatcaacatctttc
gttgtgtctaattttgaagtt	ggaatccgcaaagagtttttc	atgtgtgaatattgtgtgcac	catcccaatcttgagattcg
attgaacagaaccatcttcaa	ttccttacacttcgtttcaag	actgctcattaagacctaagt	agggcatagtttcaatttcc
ttttgttgataatggtcagct	gaaagctcttgtaattgctgc	aataatcatccagtcaggtct	aagctcttttgacgatcca
agactggaccatcaccaattg	tcttccaattgctgattatga	cggaggaggaacaggtaagac	ccaatcggtaagtattccac
aagtaatggttgtctggtaac	aactagaaacctgcttgatgg	accgaaatactaggacggaca	aacaaggcagcaatgccaac
ggataaacgagattgagtgga	gcatttatttttttccagttca	ttaaatcatcttcgccacgac	ccatccgccatatatacaaa
ctctcttttcgtttggatctt	cgctaatttgaagacgttgct	aacgttggcatttgcttttat	tcggcttgaagaatttcctc
aattctaacaagaccatgtgg	cgcagcttttaatttttccaa	ttcagagacaatatgcgcagg	tcataggattcgggtaagca
ggtaataccagcagcagtaac	gatagcttttcaattcgttct	tggcaacatggaactgaagta	agtcggcttttgagatacga
ttgtacaattcatccatacca	ccttgagaatttcaacattcc	cgctatttcattgtccatata	ctgttgggaagggggataag
	ttggactccaaatcgtttttc	tctgccagatttttgtaaagc	ctttttggaggcatacttct
	aaccttatccctatattcttc	cgtgcacggatacttttaaga	aatcttcatcagcgtcatcg
	tcattttcgagttcaagggta	gagctgaaaaatcaacccgtt	gaattatcggcgactcgttt
	gggttggtaactcgttagtaa	aatttggatcaccggtaatca	tatgcatctggcaatcttca
	acgagtttgtttgaaacagct	aacaccgagttcatctaacga	atcggcaagtagctcaacat
	ttagcattggtattctgtaga	agtgtcttagcaatactacgt	atattactccgactcatggt
	taaactagaaacgcgctctcc	aggtgtaacagtttcaggata	

Table S4: Primers for quantitative PCR

Target	Forward	Reverse
act1	CCAAATCCAACCGTGAGAAGA	GTACGACCAGAGGCATACAAAG
cdc2	GGTATCGTGCTCCTGAAGTATTG	CAGAGTCACCGGGAAATAATGG
mad1	CCTAATGGGAGTGTTCGTGTTA	CCTGATGGATTACCAACCAATTTC
mad2	TTAGAGCGGTGGCAGTTTAAT	CTCGCAGTTCATCTTCTTTGTTG
mad3	CGGATGGTTCTGGAAAGGAT	CTACCCAAGAAGTAGCCGATATG
bub1	TGTCACCAGCTATGCCTAAAG	TCGTGGCTACCGGATTACTA
ymEGFP	TGAAGGTGAAGGTGATGCTAC	CTAAGGTTGGCCATGGAACT

References

1. M. Saint, F. Bertaux, W. Tang, X.-M. M. Sun, L. Game, A. Köferle, J. Bähler, V. Shahrezaei, S. Marguerat, Single-cell imaging and RNA sequencing reveal patterns of gene expression heterogeneity during fission yeast growth and adaptation. *Nature microbiology*. 4, 480–491 (2019).

2. X.-M. Sun, A. Bowman, M. Priestman, F. Bertaux, A. Martinez-Segura, W. Tang, C. Whilding, D. Dormann, V. Shahrezaei, S. Marguerat, Size-Dependent Increase in RNA Polymerase II Initiation Rates Mediates Gene Expression Scaling with Cell Size. *Curr Biol* (2020), doi:10.1016/j.cub.2020.01.053.

3. J.-H. Roe, R. R. Burgess, M. T. Record, Temperature dependence of the rate constants of the Escherichia coli RNA polymerase-λPR promoter interaction Assignment of the kinetic steps corresponding to protein conformational change and DNA opening. *J Mol Biol*. 184, 441–453 (1985).

4. R. E. Kingston, M. R. Green, Modeling eukaryotic transcriptional activation. *Curr Biol*. 4, 325–332 (1994).

5. L. J. Friedman, J. Gelles, Mechanism of Transcription Initiation at an Activator-Dependent Promoter Defined by Single-Molecule Observation. *Cell*. 148, 679–689 (2012).

6. J. Lloyd-Price, S. Startceva, V. Kandavalli, J. G. Chandraseelan, N. Goncalves, S. M. D. Oliveira, A. Häkkinen, A. S. Ribeiro, Dissecting the stochastic transcription initiation process in live Escherichia coli. *Dna Res Int J Rapid Publ Reports Genes Genomes*. 23, 203–214 (2016).

7. G. T. Booth, I. X. Wang, V. G. Cheung, J. T. Lis, Divergence of a conserved elongation factor and transcription regulation in budding and fission yeast. *Genome Research*. 26, 799–811 (2016).

8. G. T. Booth, P. K. Parua, M. Sansó, R. P. Fisher, J. T. Lis, Cdk9 regulates a promoter-proximal checkpoint to modulate RNA polymerase II elongation rate in fission yeast. *Nature communications*. 9, 543 (2018).

9. L. Core, K. Adelman, Promoter-proximal pausing of RNA polymerase II: a nexus of gene regulation. *Genes & Development* (2019), doi:10.1101/gad.325142.119.

10. T. Filatova, N. Popovic, R. Grima, Statistics of Nascent and Mature RNA Fluctuations in a Stochastic Model of Transcriptional Initiation, Elongation, Pausing, and Termination. *B Math Biol*. 83, 3 (2021).

11. D. R. Larson, D. Zenklusen, B. Wu, J. A. Chao, R. H. Singer, Real-Time Observation of Transcription Initiation and Elongation on an Endogenous Yeast Gene. *Science*. 332, 475–478 (2011).

12. Z. Cao, T. Filatova, D. A. Oyarzún, R. Grima, A stochastic model of gene expression with polymerase recruitment and pause release. *Biophys J* (2020), doi:10.1016/j.bpj.2020.07.020.

13. S. Braichenko, J. Holehouse, R. Grima, Distinguishing between models of mammalian gene expression: telegraph-like models versus mechanistic models. *J Roy Soc Interface*. 18, 20210510 (2021).

14. R. Karmakar, A. K. Das, Effect of transcription reinitiation in stochastic gene expression. *J Statistical Mech Theory Exp*. 2021, 033502 (2021).

15. D. Cao, R. Parker, Computational modeling of eukaryotic mRNA turnover. *Rna*. 7, 1192–1212 (2001).

16. C. Deneke, R. Lipowsky, A. Valleriani, Complex Degradation Processes Lead to Non-Exponential Decay Patterns and Age-Dependent Decay Rates of Messenger RNA. *Plos One*. 8, e55442 (2013).

17. D. Schnoerr, G. Sanguinetti, R. Grima, Approximation and inference methods for stochastic biochemical kinetics—a tutorial review. *J Phys Math Theor*. 50, 093001 (2017).

18. J. Elf, M. Ehrenberg, Fast Evaluation of Fluctuations in Biochemical Networks With the Linear Noise Approximation. *Genome Res*. 13, 2475–2484 (2003).

19. J. Peccoud, B. Ycart, Markovian Modeling of Gene-Product Synthesis. *Theor Popul Biol*. 48, 222–234 (1995).

20. W. Shao, J. Zeitlinger, Paused RNA polymerase II inhibits new transcriptional initiation. *Nature Genetics*. 49, 1045–1051 (2017).

21. S. Gressel, B. Schwalb, T. M. Decker, W. Qin, H. Leonhardt, D. Eick, P. Cramer, CDK9-dependent RNA polymerase II pausing controls transcription initiation. *Elife*. 6, e29736 (2017).

22. H. Xu, S. O. Skinner, A. M. Sokac, I. Golding, Stochastic Kinetics of Nascent RNA. *Phys Rev Lett*. 117, 128101 (2016).

23. T. Zhou, J. Zhang, Analytical Results for a Multistate Gene Model. *Siam J Appl Math*. 72, 789–818 (2012).

24. J. K. Pritchard, M. T. Seielstad, A. Perez-Lezaun, M. W. Feldman, Population growth of human Y chromosomes: a study of Y chromosome microsatellites. *Mol Biol Evol*. 16, 1791–1798 (1999).

25. T. Toni, D. Welch, N. Strelkowa, A. Ipsen, M. P. H. Stumpf, Approximate Bayesian computation scheme for parameter inference and model selection in dynamical systems. *J Roy Soc Interface*. 6, 187–202 (2008).

26. D. T. Gillespie, Exact stochastic simulation of coupled chemical reactions. *J Phys Chem*. 81, 2340–2361 (1977).

27. H. Jeffreys, *Theory of Probability* (Oxford University Press, ed. 3rd, 1998; https://global.oup.com/academic/product/theory-of-probability-9780198503682).

28. J. M. Mitchison, J. Creanor, Further measurements of DNA synthesis and enzyme potential during cell cycle of fission yeast Schizosaccharomyces pombe. *Exp Cell Res*. 69, 244–247 (1971).

29. J. O. Patterson, M. Swaffer, A. Filby, An Imaging Flow Cytometry-based approach to analyse the fission yeast cell cycle in fixed cells. *Methods*. 82, 74–84 (2015).

30. U. T. Eden, M. A. Kramer, Drawing inferences from Fano factor calculations. *J Neurosci Meth*. 190, 149–152 (2010).
